# tRNA-derived small RNA dataset in multiple organs of intrauterine growth-restricted pig

**DOI:** 10.1038/s41597-023-02715-w

**Published:** 2023-11-10

**Authors:** Ma Jianfeng, Gan Mailin, Yang Yiting, Chen Lei, Zhao Ye, Niu Lili, Wang Yan, Zhang Shunhua, Wang Jingyong, Zhu Li, Shen Linyuan

**Affiliations:** 1 Farm Animal Genetic Resources Exploration and Innovation Key Laboratory of Sichuan Province, Sichuan Agricultural University, Chengdu, China; 2 Key Laboratory of Livestock and Poultry Multi-omics, Ministry of Agriculture and Rural Affairs, College of Animal and Technology, Sichuan Agricultural University, Chengdu, China; 3 Chongqing Academy of Animal Science, Chongqing, China

**Keywords:** RNA sequencing, Agricultural genetics, Gene expression

## Abstract

Intrauterine growth restriction (IUGR) impairs neonatal weight and causes multiple organ dysplasia. IUGR not only threatens human health but is also a significant constraint to the development of animal husbandry. However, the molecular mechanism underlying IUGR remains to be further elucidated. tRNA-derived small RNA (tsRNAs) is a regulative non-coding RNA, which has recently been reported to correlate with the onset and progression of several diseases. In this study, we investigated the tsRNAs expression profiles of IUGR pigs. A tsRNAs dataset for multiple organs in normal and IUGR pigs was generated, including muscle, liver, spleen and intestine. We further analyzed the characteristics of tsRNAs in different organs of pigs, and KEGG pathway analysis was performed to investigate possible pathways involved. This dataset will provide valuable information for further exploring the molecular mechanism of IUGR formation.

## Background & Summary

Intrauterine growth restriction (IUGR) broadly refers to a fetus’s slow growth and development caused by adverse factors, which usually induces low weight of newborns and multiple hypoplastic organs^1^. IUGR remains an intractable public health concern worldwide and a major problem restricting animal husbandry development^2,3^. As a multiparous mammalian animal, pigs have exhibited a naturally high incidence of IUGR^4^. IUGR permanently negatively affects mortality, postnatal growth and development for newborns. Available research shows that IUGR piglets exhibit abnormal development features, including disrupted muscle development^5,6^, immune dysfunction^7^, insulin resistance^8^, abnormal glucolipid metabolism^9^ and other diseases. These abnormal physiological changes involve the dysplasia of multiple tissues and organs. Hence, exploring the molecular mechanisms of multiple organs is vitally essential to deepen the understanding of IUGR.

In epigenetics, non-coding RNA-dependent mechanisms are essential for gene expression regulation. Recently, a tRNA-derived small non-coding RNA (tsRNAs) have been identified by high-throughput RNA sequencing^10^. tsRNAs are produced by specific nuclease cutting different sites of parental tRNA. Several nucleases, such as angiogenin, Dicer, RNase P, RNase Z, and RNase L, have been shown to cleavage tRNAs^11^. tsRNAs can be categorized into several subtypes, including tRF-1, tRF-2, tRF-3, tRF-5, tiRNA-3, tiRNA-5 based on the break site of parental tRNAs^12^. In early studies, tsRNAs were considered solely a tRNA degradation product^13^. Many studies suggests that this novel ncRNA has several important functions, including ribosome biogenesis regulation^14^, intergenerational inheritance^15^, RNA silencing^16^, and translational regulation^17^. tsRNAs are widely involved in various biological processes through the above mechanisms, such as cell proliferation, migration, apoptosis, differentiation, and cell cycle^18,19^.

**Fig. 1:**
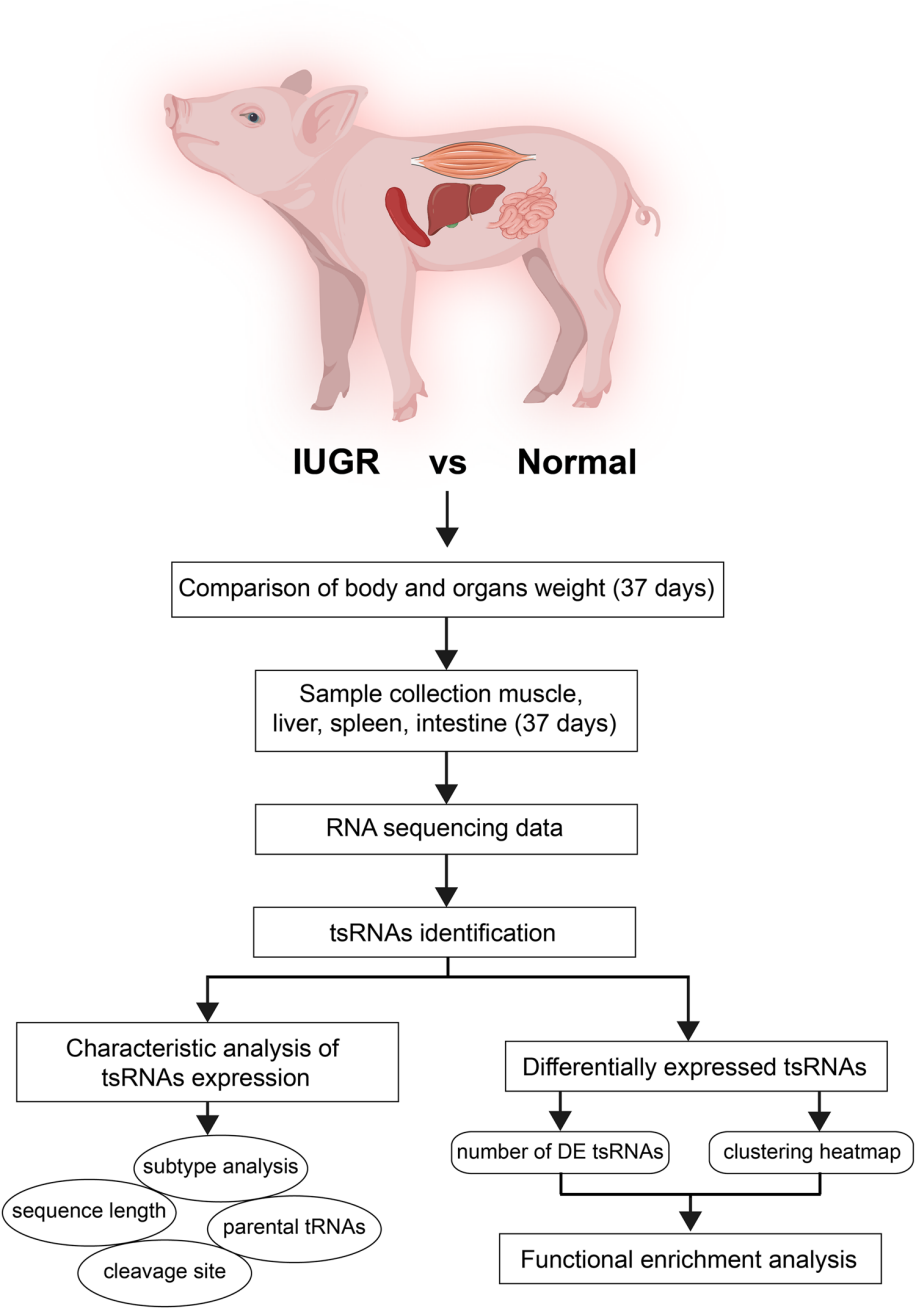
Study workflow for the main analysis.

Recently, the role of tsRNAs in the occurrence and development of diseases has attracted significant attention. However, studies about tsRNA associated with the occurrence of IUGR are still lacking. Thus, the present study aimed to characterize the expression profiles of tsRNAs in muscle, liver, spleen and intestine in the IUGR pigs model. A flow chart of this study is shown in Fig. 1.

## Methods

### Animals and sample collection

**Fig. 2:**
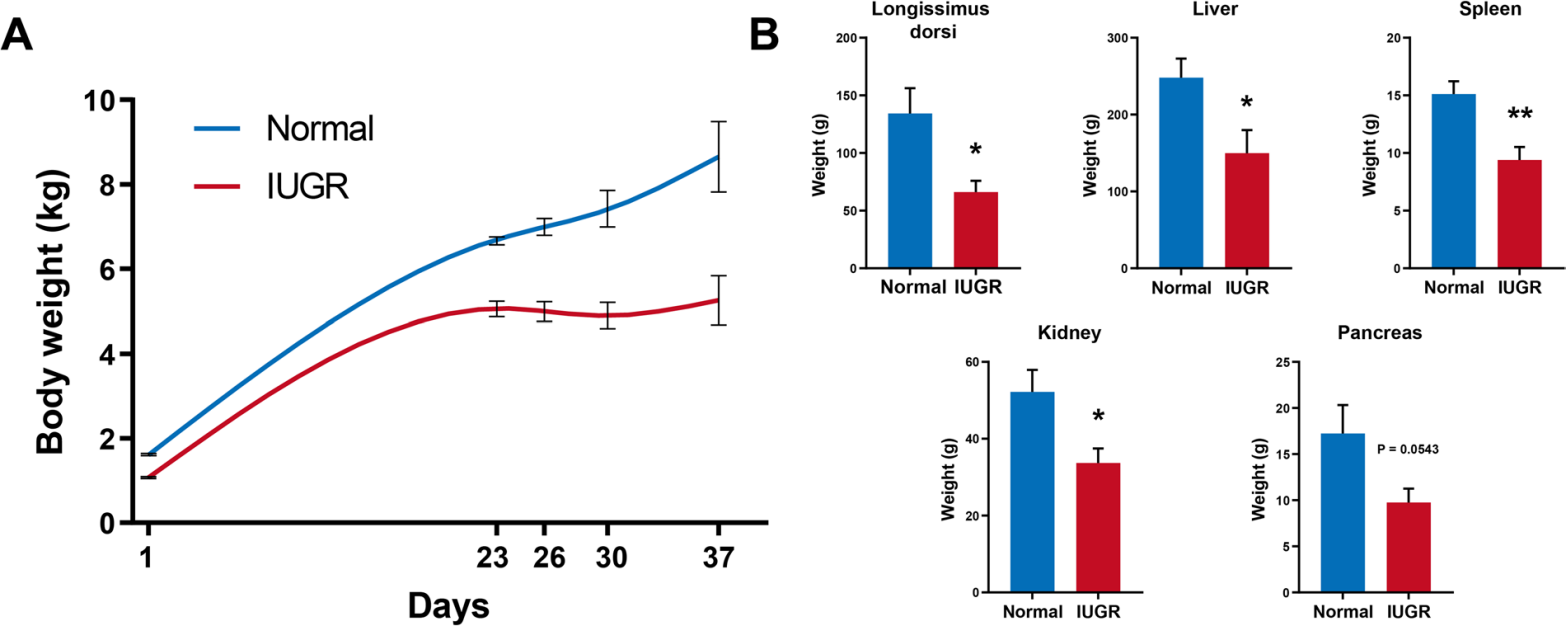
Body weight and organs weight of Normal and IUGR piglets. (**A**) The body weight of Normal and IUGR pigs at 1, 23, 26, 30, 37 days. (**B**) The Longissimus dorsi, liver, spleen, kidney, pancreas weight of Normal and IUGR pigs at 37 days. The results are represented as the mean ± SD (N = 6). *P < 0.05; **P < 0.01.

The study used 12 paternal half-sib female Duroc × Landrance × Yorkshire (DLY) piglets. They were divided into two groups according to birthweight: Normal piglets (mean birth weight 1.60 ± 0.05 g, n = 6) and IUGR piglets (mean birth weight 1.07 ± 0.04 g, n = 6). The body weight of IUGR piglets was significantly lower than the weight of normal piglets. IUGR is commonly defined as a birth weight less than two standard deviations below the normal^1^. The piglets were raised following standard commercial practice. Body weight measurements were taken at 1, 23, 26, 30, and 37 days (Fig. 2A). At 37 days, piglets were slaughtered according to a standard commercial procedure. The weight of longissimus dorsi muscle, liver, spleen, kidney and pancreas were measured separately (Fig. 2B). Longissimus dorsi muscle, liver, spleen and intestine (jejunum) samples were collected in cryopreservation tubes and stored at −80 °C until used.

### RNA extraction and library construction

Tissue samples were ground in liquid nitrogen. The number of samples per group was three. We selected the three lightest piglets in the IUGR group and the three heaviest piglets in the normal group to extract RNA. Each sample was added 1 ml RNAiso reagent (TaKaRa, Japan) to extract the total RNA according to the manufacturer’s instructions. Isolated total RNA was measured concentrations and purities using the NanoDrop 2000 spectrophotometer (Thermo Scientific, USA). The absorbance ratio of the sample at 260 nm and 280 nm is used to evaluate RNA purity. RNA samples with a ratio between 1.8 and 2.0 are used for sequencing analysis. RNA modifications are abundant in tsRNAs and interfere with small RNA-seq library construction. Before library preparation, the detailed processing flow of total RNA is as follows: 3′ - aminoacyl (charged) deacylation to 3′ –OH for 3′ adaptor ligation, 3′ -cP (2′, 3′ -cyclic phosphate) removal to 3′ -OH for 3′ adaptor ligation, 5′ -OH (hydroxyl group) phosphorylation to 5′ -P for 5′ adaptor ligation, m1A and m3C demethylation for efficient reverse transcription. The total RNA from each sample was pretreated and then utilized to prepare the tsRNA-seq library. NEBNext® Multiplex Small RNA Library Prep Set for Illumina (NEBNext®, USA) was used for library construction. Library construction steps were carried out as follows. Firstly, the RNA was ligated to 3′ and 5′ small RNA adapters. Next, cDNA was synthesized from the ligated RNA using Illumina’s proprietary reverse transcription (RT) primers and amplification primers. Subsequently, PCR amplification was performed to generate fragments ranging in size from 134–160 bp, which were extracted and purified from the polyacrylamide gel electrophoresis (PAGE) gel. Finally, the completed libraries were quantified using the Agilent 2100 Bioanalyzer to determine the concentration and quality of the libraries. The purified libraries were mixed in equal amounts and then used for sequencing.

### Sequencing procedures

The libraries were denatured with 0.1 M NaOH to generate single-stranded DNA molecules and diluted to a loading volume of 1.3 ml and loading concentration of 1.8pM. Diluted libraries were loaded onto reagent cartridges and forwarded to sequencing run on Illumina NextSeq500 system using NextSeq 500/550 V2 kit (#FC-404-2005, Illumina), according to the manufacturer’s instructions. For standard small RNA sequencing on Illumina NextSeq instrument, the sequencing type is 50 bp single-read.

### Data analysis

Raw sequence data in FASTQ format generated from the Illumina NextSeq500 sequencing platform were used for further analysis. First, the FastQC (v0.11.7) was used to assess the quality scores of sequencing reads (Table 1). tRNA cytoplasmic sequences were downloaded from the Genomic tRNA Database (GtRNAdb)^20^. The reference genome used was Sscrofa11.1. tRNA sequences of mitochondria were predicted with tRNAscan-SE^21^ software. Raw sequence were trimmed 5′, 3′ -adaptor sequence and discarded reads (length < 14nt or length > 40nt) to generate trimmed reads using Cutadapt. Trimmed reads were aligned to mature tRNA sequences, allowing onely one mismatch, and then reads that did not map were aligned to precursor tRNA sequences, allowing one mismatch with Bowtie software.

**Table 1:** Sequence reads information.

Sample	TotalRead	TotalBase	BaseQ30(%)	BaseQ30(%)
N_muscle_1	8458063	431361213	403991974	93.66
N_muscle_2	7594170	387302670	363591378	93.88
N_muscle_3	8711338	444278238	415958204	93.63
I_muscle_1	7114889	362859339	340631172	93.87
I_muscle_2	8740452	445763052	418426914	93.87
I_muscle_3	7685355	391953105	366161390	93.42
N_liver_1	9850607	502380957	466381284	92.83
N_liver_2	9633566	491311866	457830177	93.19
N_liver_3	7965336	406232136	376132343	92.59
I_liver_1	9675882	493469982	456708223	92.55
I_liver_2	7027261	358390311	332412961	92.75
I_liver_3	6173189	314832639	291492450	92.59
N_spleen_1	10899175	555857925	516461077	92.91
N_spleen_2	8803120	448959120	416306922	92.73
N_spleen_3	8532766	435171066	404191225	92.88
I_spleen_1	7892275	402506025	373796577	92.87
I_spleen_2	8072638	411704538	381167847	92.58
I_spleen_3	7523623	383704773	356944901	93.03
N_intestine_1	9113859	464806809	429251217	92.35
N_intestine_2	9349474	476823174	441933839	92.68
N_intestine_3	9636017	491436867	454479591	92.48
I_intestine_1	9059508	462034908	427921339	92.62
I_intestine_2	9196974	469045674	432253819	92.16
I_intestine_3	9223586	470402886	437215188	92.94

TotalRead: Raw sequencing reads after quality filtering. TotalBase: Number of bases after quality filtering. BaseQ30(%): Number of bases of Q score more than 30 after quality filtering. BaseQ30(%): The proportion of bases (Q ≥ 30) number after quality filtering.

The expression level of each tsRNA is evaluated using sequencing counts and is normalized as counts per million of total reads (CPM). The count and CPM of tsRNAs can be calculated with the following formula:

$${\rm{Count}}=\mathop{\sum }\limits_{i=1}^{n}\frac{{c}_{i}}{{m}_{i}},\quad \quad {\rm{CPM}}=\frac{1{0}^{6}Count}{N}$$

i: the i-th read aligned to the tsRNA region; n: the number of the reads aligned to the tsRNA region; c_i_: the count of the i-th read; m_i_: the number of tsRNA generated from the i-th read (m_i_ possibly occur great than one, only when allowing for more than 1 mismatch); N: the total number of reads mapped onto all of the mature or precursor tRNA. The obtained counts and CPM data of tsRNA were used for subsequent analysis.

### Characteristics of tsRNAs expression profile

**Fig. 3:**
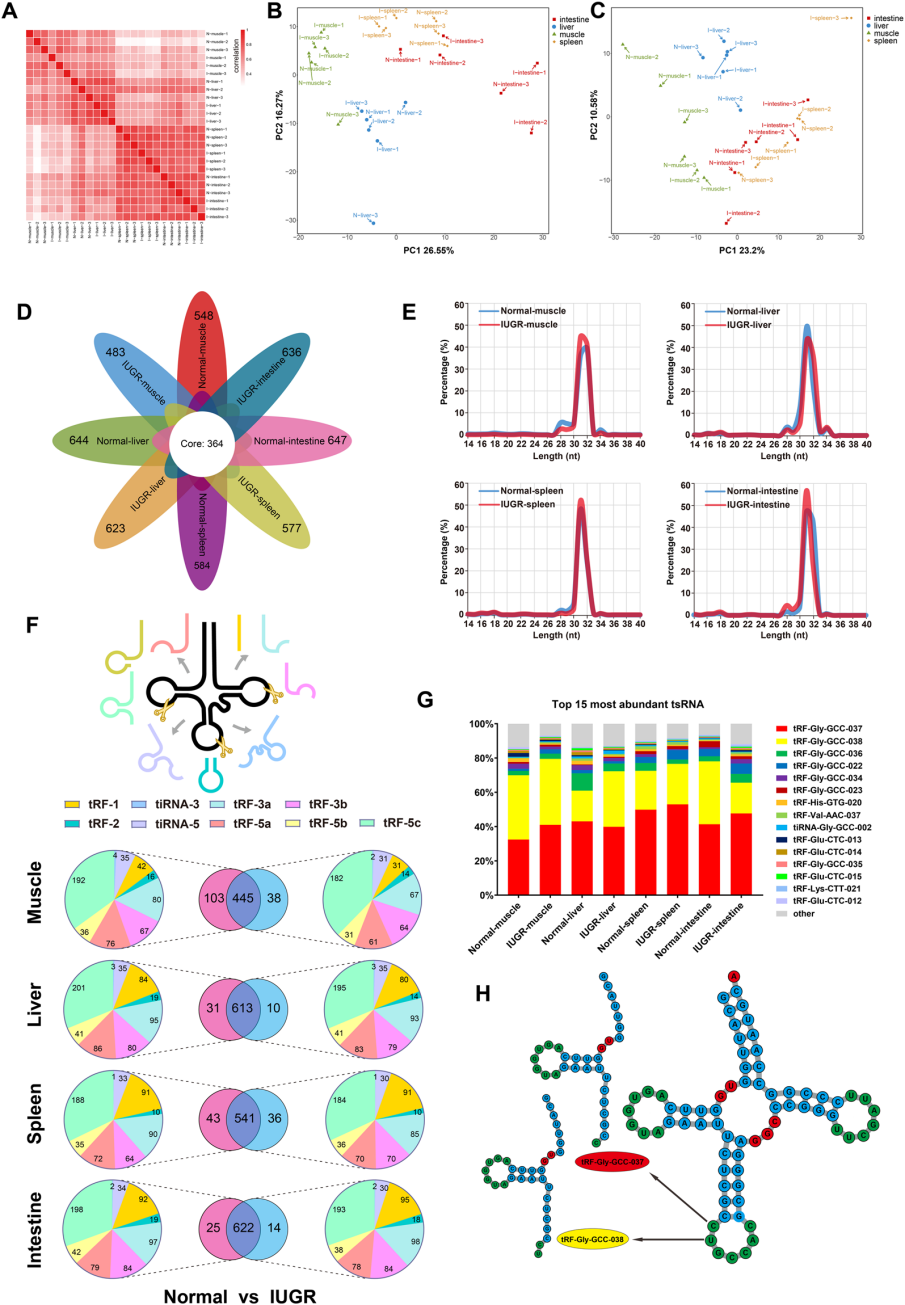
Analysis of tsRNAs characteristics. (**A**) Spearman’s correlations coefficients between all samples. Principal Component Analysis (PCA) based on read counts (**B**) and CPM value (**C**) of samples. (**D**) numbers of tsRNAs for each tissue type. (**E**) tsRNAs length distribution in muscle, liver, spleen and intestine of normal and IUGR pigs. (**F**) Venn diagram summarizing tsRNAs number and type of Normal and IUGR piglets. (**G**) Relative abundance of the top most abundant 15 tsRNAs. (**H**) The generation position of tRF-Gly-GCC-037/038 derived from tRNA-Gly-GCC.

Based on the tsRNAs expression level (CPM), we evaluated Spearman’s correlations coefficients between 24 samples (Fig. 3A). Principal Component Analysis was performed based on read counts (Fig. 3B) and CPM (Fig. 3C) of tsRNAs for each sample. The number of tsRNAs identified from each group was depicted in the petal diagram, and 364 core tsRNAs were shared among all groups (Fig. 3D). The length distribution of the identified tsRNAs was analyzed (Fig. 3E). The majority of tsRNAs were 31–32 nt in length. According to the cleavage site of parental tRNAs, tsRNAs were classified into 9 subtypes, as shown in Fig. 3F. Conventional and specifically expressed tsRNAs between normal and IUGR groups are depicted in the Fig. 3F Venn diagram. The pie chart shows the number of each group’s tsRNAs subpopulation. We further analyzed the percentages of per tsRNAs in each group. The percentage of the top 15 tsRNAs was also computed in all groups (Fig. 3H). Among them, tRF-Gly-GCC-037/038 was the highest in abundance, the sum of two tsRNAs exceeded the 60%. Interestingly, the top 6 tsRNAs all originate from the same tRNA-Gly-GCC. Figure 3H illustrates the sequence of tRF-Gly-GCC-037/038 and their parental tRNA-Gly-GCC cleavage site.

**Fig. 4:**
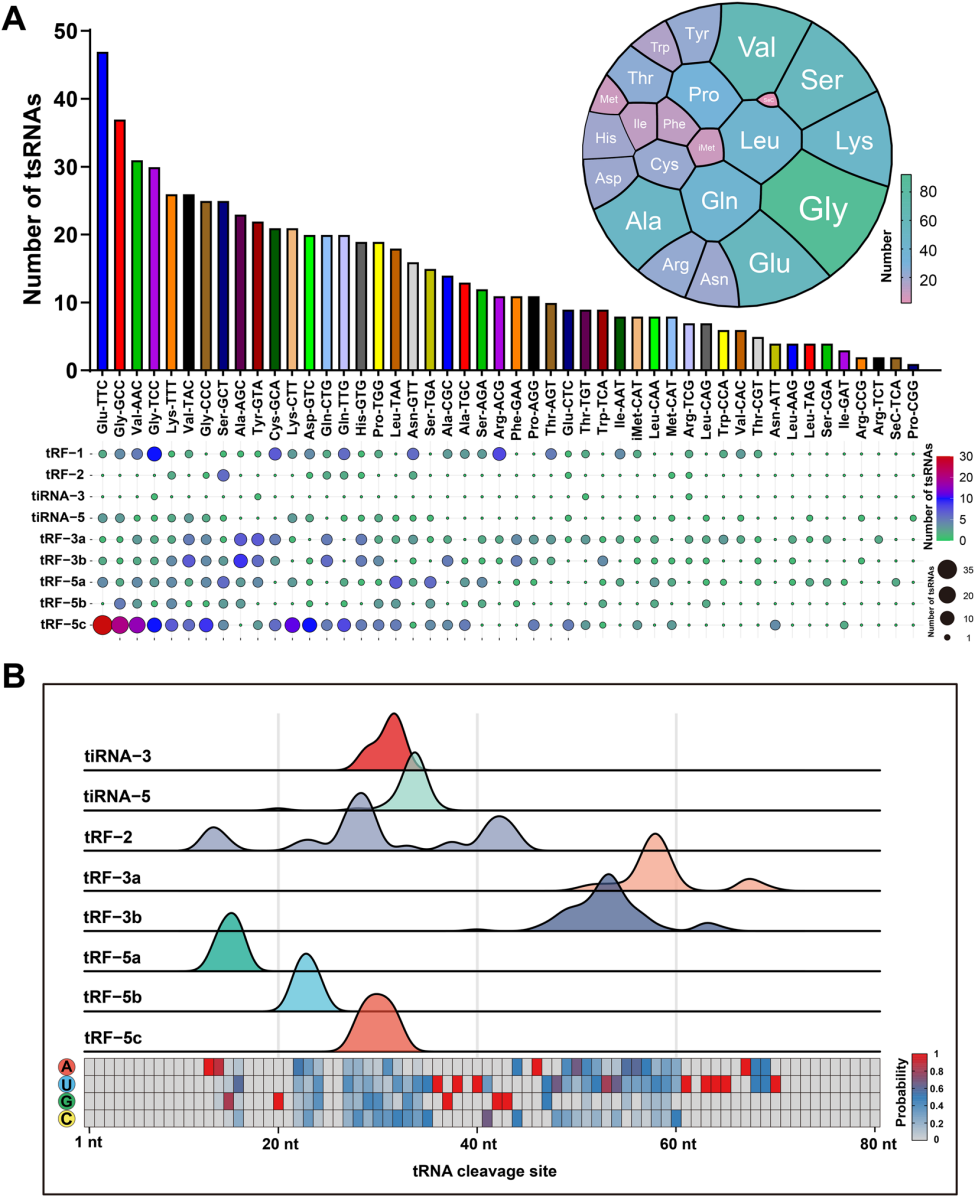
Analysis of parental tRNA. (**A**) Statistics of amino acids corresponding to parent tRNA of tsRNAs. The bar chart represents the number of tsRNAs corresponding to different tRNAs. The spherical plot represents the number of tsRNAs corresponding to different amino acids. The bubble chart represents the number of different subtypes tsRNAs. (**B**) Cleavage position of each type of tsRNAs and base characteristics. The peak represents the probability of cleavage site. The sum of peak areas for each subtype is 1.

We further analyzed the characteristics of tsRNAs identified in four tissues of pigs. Figure 4A demonstrates the tRNA types from which the tsRNAs originate and the number of tsRNA subtypes. It indicated that the tRNA-Glu-TTC produced the most significant number of tsRNAs. As shown in both Figs. 3F, 4A diagrams, tRF-5c was the most abundant tsRNA subtype in any one sample. Moreover, the tRNA cleavage sites corresponding to each subtype were analyzed in Fig. 4B. We calculated the proportion of four bases in each break site of tRNA in Fig. 4B lower panel.

### Identification of differentially expressed tsRNAs

**Fig. 5:**
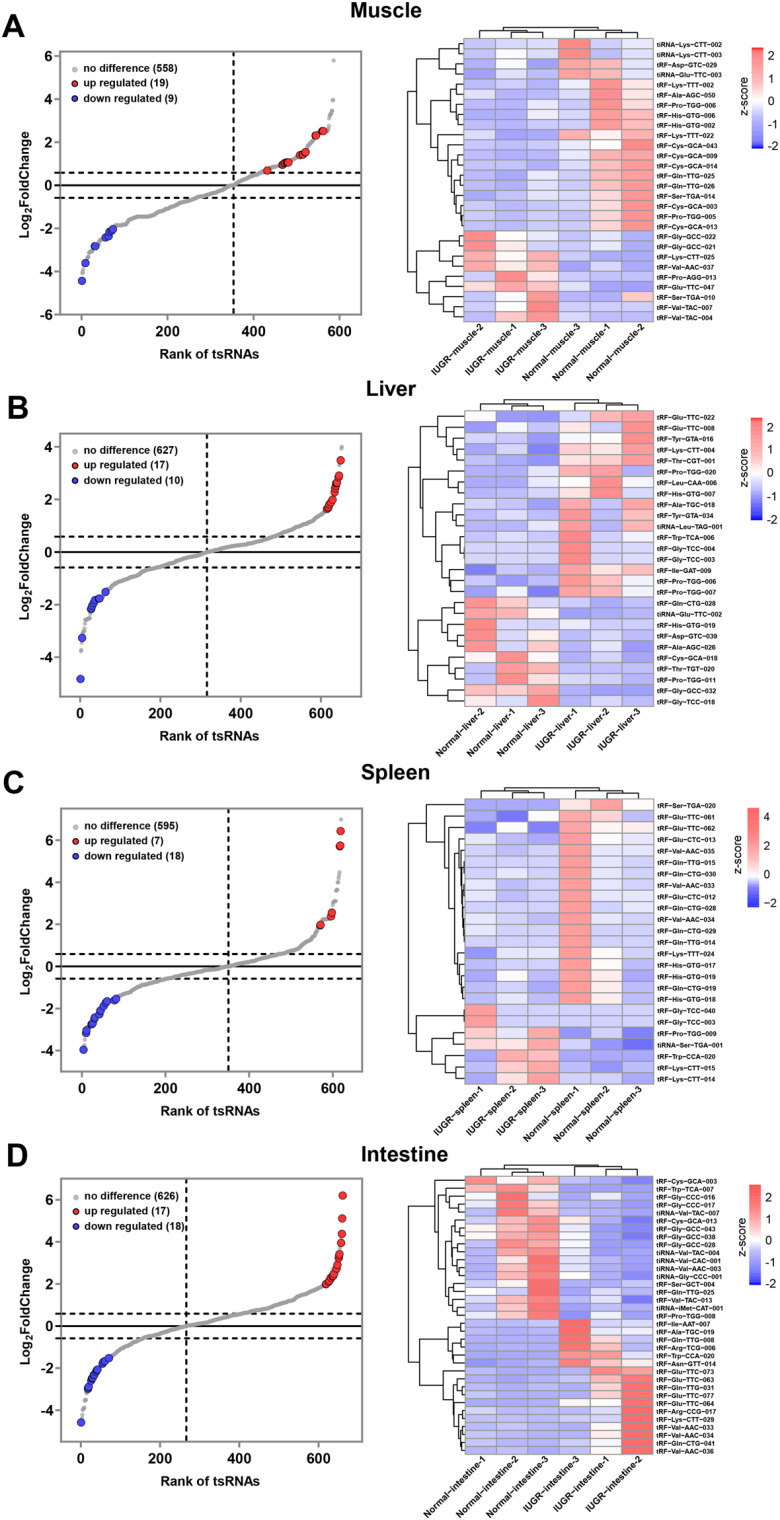
Analysis of differentially expressed tsRNAs. (**A**–**D**) represent muscle, liver, spleen and intestine, respectively. The left panel is the tsRNA rank plot, the right panel is the heatmap of differentially expressed tsRNAs. The heatmap is based on the expression level of tsRNA (CPM) and used Z-Score for standardization.

Differentially expressed tsRNAs analyses were performed with R package edgeR. The P-value of the exact test was calculated by negative binomial distribution. Then, multiple testing using a FDR was applied to obtain the Q-values. No differentially expressed tsRNAs were found with FDR correction. The threshold for screening differentially expressed tsRNAs was the absolute fold change > 1.5 and *P*-value < 0.05. Differentially expressed tsRNAs in four tissues between normal and IUGR groups were visualized according to fold change and *P*-value. The red circle represents up-regulated tsRNAs, and blue circle indicates down-regulated tsRNAs (Fig. 5 left panel). Heat map showing differentially expressed tsRNAs clustering for each tissues (Fig. 5 right panel).

### Functional enrichment analysis

**Fig. 6:**
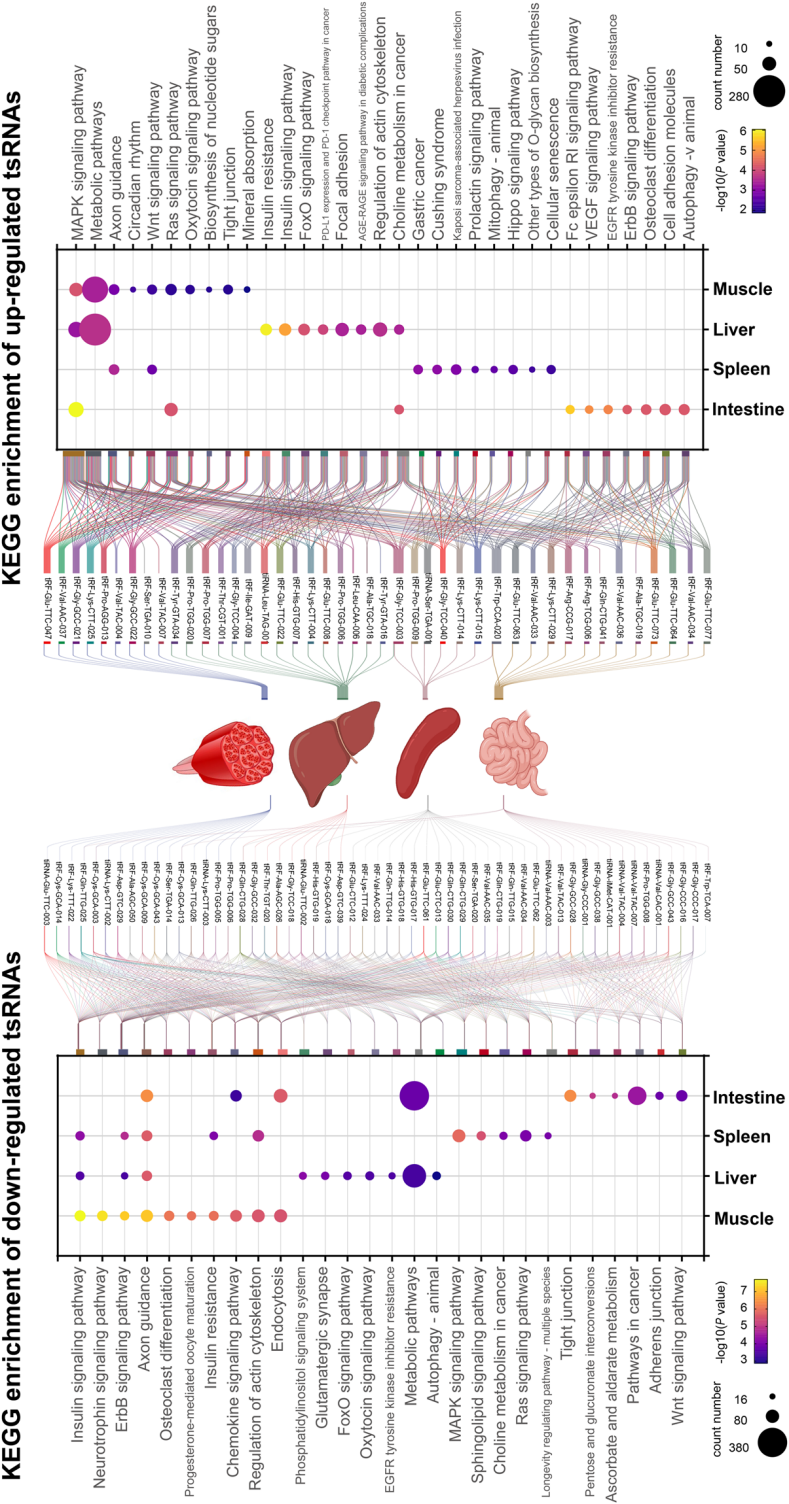
KEGG pathway enrichment analysis of up-regulated and down-regulated.

Multiple recent studies have demonstrated that tsRNAs have similar regulation mechanisms to microRNAs. Thus, we used the publicly available miRanda and TargetScan tools to predict the target genes of tsRNAs. Targetscan software threshold was set at 50 (context score percentile), and miRanda was set with a maximum binding free energy of less than −20. Those genes predicted jointly in miRanda and TargetScan were used as the target genes of tsRNAs for the Kyoto Encyclopedia of Genes and Genomics (KEGG) analysis. All up-regulated and down-regulated tsRNAs in four tissues were performed KEGG pathway enrichment analysis, respectively. The top 10 pathways for each tissue are shown in Fig. 6. Up-regulated tsRNAs were mainly enriched in the MAPK signaling pathway and metabolic pathway. Down-regulated tsRNAs were mainly enriched in the insulin signaling pathway and ErbB signaling pathway. We also constructed the relationship between these pathways and up-regulated and down-regulated tsRNAs.

### Statistical analyses.rson

Results in Fig. 2B were represented as means ± SD. Significant differences between normal and IUGR group were determined by the unpaired t-tests. *P*-values < 0.05 (*) represent significant difference. *P*-values < 0.01 (**) represent highly significant difference.

## Data Records

The RNA-Seq raw data were deposited in the NCBI Sequence Read Archive (SRA) database under the accession number PRJNA974817^22^ and PRJNA800654^23^. The tRNA sequences, results of differential expression analysis and the functional enrichment analysis are stored in figshare^24^.

## Technical Validation

### Sequencing data quality assessment

Raw data were obtained by Illumina platform. FastQC software (v0.11.7) was used to assess quality scores of raw data for each samples. Quality score Q was used to predict the probability of base-calling error (*P*): Q = −10*log*_10_(*P*). Q30 means the incorrect base calling probability to be 0.001 or 99.9% base calling accuracy. Table 1. show the proportion of bases (Q ≥ 30) number after quality filtering.

### Validation of experimental sample

Pearsons correlation coefficient analysis was performed on all 24 samples. Strong correlations were seen between samples from the same tissue type (Fig. 3A). Principal component analysis (PCA) was also performed with all samples and the distances between the sample points represent the similarity of samples. Obviously, the distance between samples from the same tissue type is closer (Fig. 3B,C).

## Data Availability

Raw sequencing data were analyzed using publicly available bioinformatics softwares. We used common data analysis software packages and no custom code was created. Software tools used are as follows: FastQC: v0.11.7, https://www.bioinformatics.babraham.ac.uk/projects/fastqc/ Bowtie: v1.2.2, https://bowtie-bio.sourceforge.net/index.shtml GtRNAdb: http://gtrnadb.ucsc.edu/ tRNAscan-SE: v2.0, http://lowelab.ucsc.edu/tRNAscan-SE/ Cutadapt: v1.17, https://github.com/marcelm/cutadapt/ edgeR: v3.24.3, https://bioconductor.org/packages/release/bioc/html/edgeR.html R software: v3.5.1, https://www.r-project.org/ OmicStudio tools (https://www.omicstudio.cn/tool) was used for prediction of target genes. GraphPad Prism 9 (GraphPad Software Inc., USA) was used for statistical analyses and data visualization.
