# Author Correction: tRNA-derived small RNA dataset in multiple organs of intrauterine growth-restricted pig

**DOI:** 10.1038/s41597-024-03987-6

**Published:** 2024-10-11

**Authors:** Ma Jianfeng, Gan Mailin, Yang Yiting, Chen Lei, Zhao Ye, Niu Lili, Wang Yan, Zhang Shunhua, Wang Jingyong, Zhu Li, Shen Linyuan

**Affiliations:** 1 Farm Animal Genetic Resources Exploration and Innovation Key Laboratory of Sichuan Province, Sichuan Agricultural University, Chengdu, China; 2 Key Laboratory of Livestock and Poultry Multi-omics, Ministry of Agriculture and Rural Affairs, College of Animal and Technology, Sichuan Agricultural University, Chengdu, China; 3 Chongqing Academy of Animal Science, Chongqing, China

Correction to: *Scientific Data* 10.1038/s41597-023-02715-w, published online 10 November 2023

In this article the author name Yang Yiting was incorrectly written as Yang Yitang. The original article has been corrected.

